# Randomized Feasibility Trial of Routine Versus Selective Transesophageal Echocardiography During Isolated Coronary Artery Bypass Grafting

**DOI:** 10.1016/j.jacadv.2026.103030

**Published:** 2026-07-20

**Authors:** Emily J. MacKay, Bo Zhang, Marisa N. Konig, Kelli N. Cook, Waleed A. Mujib, Jeremy D. Kukafka, Marisa Cevasco, Chase R. Brown, Jay Giri, Wilson Y. Szeto, ECHO Pilot Trial Investigators, Lourdes F. Al Ghofaily, Yianni Augoustides, Maurer Biscotti, Chase Brown, Alessandra I. Cardi, Marisa Cevasco, Navdeep K. Chahal, Joseph A. Colao, Holly E. Corkill, Jared Feinman, Emily Gordon, Jacob Gutsche, Michael Ibrahim, Jiri Horak, Zev Noah Kornfield, Jeremy Kukafka, Kendall Lawrence, Regina E. Linganna, Emily J. MacKay, Tai Mandelbaum, Bonnie Milas, Saumil Patel, Stuart M. Sacks, Joseph Savino, Ronak Shah, Audrey Spelde, WiIson Y. Szeto, Nabil Thalji, Asad A. Usman, William Vernick, Kelvin Wang, Stuart J. Weiss, Elizabeth Zhou, Wilson Y. Szeto

**Affiliations:** a Department of Anesthesiology and Critical Care, Perelman School of Medicine at the University of Pennsylvania, Philadelphia, Pennsylvania, USA; b Vaccine and Infectious Disease Division, Fred Hutchinson Cancer Center, Seattle, Washington, USA; c Division of Cardiovascular Surgery, Perelman School of Medicine at the University of Pennsylvania, Philadelphia, Pennsylvania, USA; d Perelman School of Medicine at the University of Pennsylvania, Philadelphia, Pennsylvania, USA; e Cardiovascular Division, Perelman School of Medicine at the University of Pennsylvania, Philadelphia, Pennsylvania, USA; f Penn’s Cardiovascular Outcomes, Quality, and Evaluative Research Center (CAVOQER), University of Pennsylvania, Philadelphia, Pennsylvania, USA; g Leonard Davis Institute of Health Economics (LDI), University of Pennsylvania, Philadelphia, Pennsylvania, USA

**Keywords:** cardiac anesthesia, coronary artery bypass grafting, intraoperative monitoring, randomized controlled trial, transesophageal echocardiography

## Abstract

**Background:**

Intraoperative transesophageal echocardiography (TEE) is used in approximately half of isolated coronary artery bypass grafting (CABG) procedures in the United States, yet its routine use remains unsupported by randomized evidence.

**Objectives:**

The objective of the study was to assess the feasibility and protocol adherence of routine vs selective intraoperative TEE strategies during isolated CABG surgery.

**Methods:**

We conducted a pragmatic, randomized feasibility trial at 2 tertiary-care hospitals within a single health system. Adults undergoing isolated CABG for whom both strategies were clinically acceptable were randomized 1:1 to TEE-by-default (routine use) or TEE-on-demand (selective use). In the selective group, TEE was performed as clinically indicated. Primary feasibility outcomes included enrollment, successful randomization, and protocol adherence. Exploratory outcomes included perioperative clinical events, patient-centered recovery measures, and TEE-related adverse events.

**Results:**

A total of 274 patients were screened and 72 were eligible. Of these, 46 (64%) consented, and 40 (56% of eligible; 87% of consented) were randomized following induction of anesthesia (20 per group). Protocol adherence was high (39/40; 98%) with 1 allocation error. In the TEE-on-demand arm, 3 of 20 patients (15%) underwent clinically triggered TEE. No deaths occurred within 90 days. Three serious adverse events were adjudicated as related to TEE exposure including 2 esophageal complications and 1 conversion to combined CABG and mitral valve replacement.

**Conclusions:**

In this pragmatic randomized feasibility trial, both TEE strategies were successfully implemented with high protocol adherence and preserved clinician and patient equipoise, supporting a multicenter randomized trial evaluating routine vs selective TEE during isolated CABG.

Intraoperative transesophageal echocardiography (TEE) is a cornerstone of modern cardiac surgery,^1^, ^2^, ^3^ with observational comparative effectiveness data supporting its use in complex procedures such as valve and proximal aortic surgery.^4^^,^^5^ In contrast, its role during isolated coronary artery bypass grafting (CABG) remains uncertain,^6^, ^7^, ^8^, ^9^, ^10^ and randomized controlled trial evidence demonstrating clinical benefit in this population is lacking. In isolated CABG, available observational studies have yielded inconsistent findings^6^, ^7^, ^8^, ^9^, ^10^ leaving the balance of potential benefit and harm unresolved.

Routine use of TEE in isolated CABG must therefore balance potential benefit against risk. Although TEE provides real-time visualization to guide management, it is an invasive procedure associated with probe-related gastroesophageal injury.^11^^,^^12^ Consistent with this evidentiary gap, professional guidelines classify TEE in isolated CABG as having “unknown usefulness” (class IIb).^13^^,^^14^ Practice patterns mirror this uncertainty: intraoperative TEE is used in approximately half of isolated CABG surgeries in the United States,^6^, ^7^, ^8^, ^9^ with substantial geographic variation (11%-91% across states),^7^ and utilization appears more strongly influenced by institutional or provider preference than by patient risk profile.^15^^,^^16^

This marked practice variation provides a setting in which randomization between accepted strategies is both clinically appropriate and ethically justified. To evaluate the feasibility of a definitive trial and to characterize real-world equipoise, we conducted the ECHO (Echocardiography in CABG and Heart Outcomes) pilot trial, a two-hospital randomized feasibility study within a single health system comparing 2 established intraoperative TEE strategies for isolated CABG: TEE-by-default and TEE-on-demand (Central Illustration). The primary objective was to assess the feasibility of enrollment, randomization, and protocol adherence. Secondary objectives included exploratory evaluation of perioperative clinical outcomes and probe-related complications to inform the design of a future multicenter pragmatic randomized controlled trial. We hypothesized that randomization between these 2 TEE strategies would be feasible and acceptable in contemporary clinical practice.

**Central Illustration:**
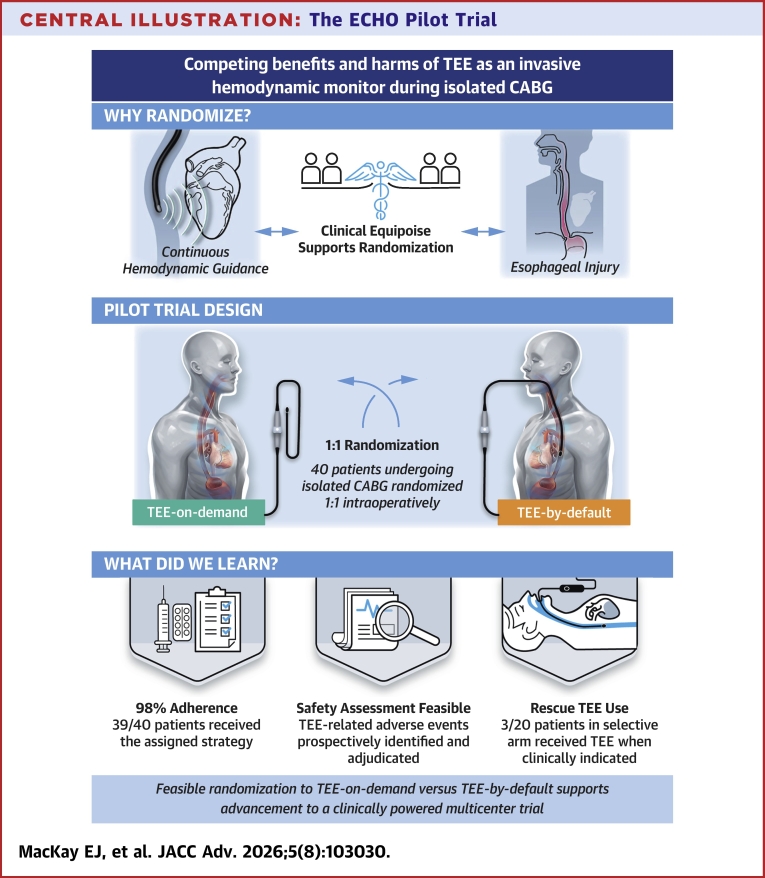
**TheECHO Pilot Trial** The ECHO (Echocardiography in CABG and Heart Outcomes) Pilot Trial randomized 40 adults undergoing isolated CABG to TEE-by-default vs TEE-on-demand. The trial demonstrated feasible intraoperative randomization, high protocol adherence, and preserved clinical equipoise, supporting advancement to a clinically powered multicenter trial evaluating routine vs selective intraoperative TEE strategies. CABG = coronary artery bypass grafting; TEE = transesophageal echocardiography.

## Methods

### Study design and setting

This prospective, randomized feasibility trial was conducted at 2 hospitals within a single health system. The protocol was approved by the University of Pennsylvania Institutional Review Board, and the trial was registered on ClinicalTrials.gov (NCT06154265) before patient enrollment. The trial was conducted in accordance with the Consolidated Standards of Reporting Trials (CONSORT) guidelines.^17^

### Participants

Eligible participants were adults (≥18 years) undergoing isolated CABG at 1 of 2 academic hospitals within a single health system. To ensure clinical equipoise and procedural homogeneity, inclusion criteria required preserved left ventricular systolic function (ejection fraction [EF] ≥50%), recent preoperative cardiac imaging evaluation (transthoracic echocardiography and coronary angiography within 1 year), and the ability to provide informed consent in English or with a certified interpreter (Supplemental Table 1). Patients were identified during outpatient surgical consultation or inpatient admission before surgery, and written informed consent was obtained before randomization.

Key exclusion criteria were prespecified to enhance safety and to focus on patients in whom both TEE strategies were clinically acceptable. Selected exclusion criteria included moderate or greater valvular heart disease, planned concomitant valve or aortic procedures, prior cardiac surgery, high-risk coronary anatomy (eg, >90% left main coronary occlusion), advanced kidney disease, severe pulmonary hypertension, presurgical mechanical circulatory support, and contraindications to TEE. Patients who developed hemodynamic instability after induction of anesthesia, as defined in the protocol, were excluded before randomization and received intraoperative TEE for clinical management. The complete list of eligibility criteria is provided in Supplemental Table 1.

Both participating hospitals are high-volume academic centers in which intraoperative TEE is commonly used during isolated CABG, except in the presence of clear contraindications.

### Randomization and blinding

Randomization was performed intraoperatively on the day of surgery after placement of invasive arterial monitoring, induction of general anesthesia, and endotracheal intubation. This timing allowed exclusion of patients who developed hemodynamic instability after induction, as defined by prespecified criteria (Supplemental Table 1), thereby preserving patient safety and maintaining clinical equipoise before treatment assignment.

Participants were randomized in a 1:1 ratio to TEE-by-default or TEE-on-demand using a computer-generated sequence with simple randomization. Allocation was concealed until assignment. Block randomization was not used because the primary objective of this feasibility trial was to assess the operational practicality of randomization and protocol adherence rather than to ensure balanced allocation within short enrollment intervals.

This was a single-blind trial. Participants were blinded to treatment assignment; however, blinding of the intraoperative clinical team was not feasible given the visible presence of TEE equipment and real-time imaging in the operating room.

### Intervention strategies

Participants randomized to the TEE-by-default arm underwent TEE probe placement immediately following endotracheal intubation and received intraoperative echocardiographic assessment before and after surgical revascularization, consistent with standard perioperative practice.

Participants randomized to the TEE-on-demand arm did not undergo planned probe placement after intubation. The TEE system remained available in the operating room, and probe placement was permitted at any point during the procedure at the discretion of the attending cardiac anesthesiologist or upon request by the attending cardiac surgeon in response to intraoperative clinical concerns (eg, unexplained hemodynamic instability, suspected ventricular dysfunction, or regional wall motion abnormalities). To preserve the pragmatic design and reflect contemporary practice, indications for triggered TEE were not protocol-mandated but were prospectively documented.

In both arms, intraoperative management was led by the attending cardiac anesthesiologist and attending cardiac surgeon per usual practice, and the randomized assignment applied only to the planned TEE monitoring strategy.

### Feasibility outcomes

The primary objective of this randomized feasibility trial was to evaluate the operational feasibility of conducting a definitive multicenter trial comparing intraoperative TEE strategies during isolated CABG. Accordingly, primary outcomes were prespecified feasibility metrics—enrollment rate, successful randomization, and protocol adherence—rather than clinical endpoints.

First, recruitment feasibility was assessed by quantifying patient flow from screening through randomization. Prespecified metrics included: the number of patients screened, the number meeting eligibility criteria (including surgeon-adjudicated presence of clinical equipoise), the number approached for participation, the number providing informed consent, and the number successfully randomized. Recruitment performance was summarized as the proportion of eligible patients who consented and the proportion who were ultimately randomized.

Second, protocol adherence was evaluated by fidelity to assigned treatment strategy. In the TEE-by-default arm, adherence was defined as successful probe placement with completion of prespecified intraoperative TEE examinations before and after revascularization. In the TEE-on-demand arm, adherence was defined as no planned probe placement after induction of anesthesia. Any TEE examinations performed in the TEE-on-demand arm were documented prospectively with their clinical rationale and were considered protocol-consistent when triggered at the discretion of the treating attending surgeons or anesthesiologists.

### Exploratory clinical outcomes

Prespecified exploratory clinical outcomes were collected to inform outcome selection, effect size estimation, and statistical power calculations for a future multicenter trial. These included postoperative laboratory markers of organ function (postoperative serum creatinine and lactate), duration of postoperative mechanical ventilation during the index hospitalization, and days alive and out of hospital at 90 days.

In addition, exploratory patient-reported gastrointestinal symptoms were assessed within 24 and 48 hours after surgery using nonvalidated binary survey items. These included sore throat, dry throat, swallowing difficulty, painful swallowing, nausea or vomiting, gastroesophageal reflux symptoms, and taste of blood. These data were collected to inform selection and refinement of patient-centered endpoints for a future trial.

Given the feasibility design and limited sample size, analyses of all exploratory outcomes were descriptive and were not intended to support formal hypothesis testing or definitive between-group inference.

### Statistical analysis

This randomized feasibility trial was not powered to detect differences in clinical outcomes. The primary objective was to evaluate feasibility of enrollment, randomization, and protocol adherence; accordingly, analyses focused on prespecified feasibility metrics. We also quantified the number of unique attending cardiac surgeons and cardiac anesthesiologists who actively participated in cases that were ultimately randomized.

All analyses were conducted according to randomized assignment (intention-to-treat). Feasibility endpoints included the number of patients screened, eligible, consented, and randomized, as well as protocol adherence within each treatment arm. These outcomes were summarized using counts and percentages, with exact binomial 95% CIs where appropriate. No formal hypothesis testing was prespecified for feasibility endpoints.

Exploratory clinical outcomes were summarized by randomized group. Continuous variables are presented as means with SDs and 95% CIS, and categorical variables as counts and percentages. Between-group differences with 95% CIs are provided to inform effect size estimation. No formal hypothesis testing was performed.

Statistical analyses were performed using Stata (version 19; StataCorp). Figures were generated using Python (Python Software Foundation).

### Protocol monitoring and safety oversight

Protocol adherence was monitored prospectively by the study team. In the TEE-by-default arm, adherence was defined as completion of both presurgical and postsurgical intraoperative TEE examinations. Any missed or incomplete examinations were classified as protocol deviations and documented with associated clinical context.

In the TEE-on-demand arm, triggered TEE use was permitted at the discretion of the attending cardiac anesthesiologist or surgeon in response to intraoperative clinical concerns and was not considered a protocol deviation. The frequency of TEE use triggered by intraoperative clinical judgment was recorded prospectively, and the indication for each examination was documented.

An independent Data and Safety Monitoring Board (DSMB) provided oversight of enrollment, feasibility metrics, and adverse events. All serious adverse events were reviewed by the DSMB. No safety-related study suspension or protocol modification was recommended during the trial.

## Results

### Participant enrollment and baseline characteristics

**Figure 1:**
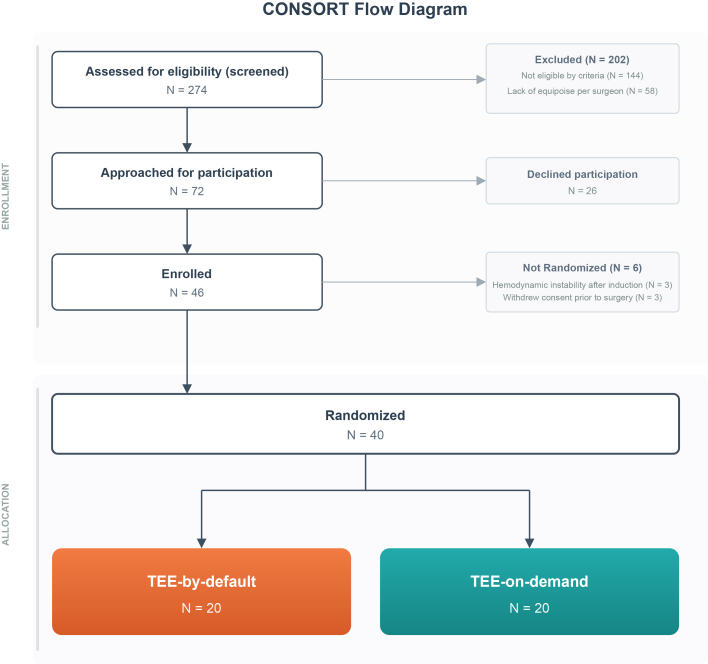
**Consolidated Standards of Reporting Trials Diagram** Flow of participants through screening, enrollment, randomization, and allocation in the randomized feasibility trial comparing TEE-by-default and TEE-on-demand strategies during isolated coronary artery bypass grafting (CABG). Of 274 patients screened, 72 were approached for participation, 46 provided consent, and 40 were successfully randomized following confirmation of intraoperative hemodynamic stability. Reasons for exclusion and nonrandomization are shown. TEE = transesophageal echocardiography.

Between January 9, 2024, and March 13, 2025, 274 patients were screened for eligibility across 2 hospitals within a single health system (Figure 1). Of these, 202 were excluded, including 144 who did not meet prespecified inclusion criteria and 58 for whom surgeon-level clinical equipoise was not present. Most screened patients were ineligible due to prespecified exclusion criteria (eg moderate or greater valvular disease, reduced EF, or high-risk coronary lesions), reflecting the higher complexity case mix at the University of Pennsylvania health system. These conservative eligibility criteria were used during the pilot phase to support surgeon and anesthesiologist trial acceptance and protocol fidelity.

Among the 72 eligible patients, 46 (64%) provided informed consent. Forty participants (56% of eligible patients; 87% of those consented) were successfully randomized following induction of anesthesia. Six consented patients were not randomized: three developed hemodynamic instability after induction and 3 withdrew consent before incision (Figure 1). Among randomized participants, 20 were allocated to TEE-by-default and 20 to TEE-on-demand. Six attending cardiac surgeons and 27 attending cardiac anesthesiologists participated in care of randomized trial patients (Supplemental Table 4).

Baseline demographic and clinical characteristics are summarized in Table 1. The study population reflected typical patients undergoing isolated CABG surgery, with a mean age of 65.4 ± 9.3 years and 88% male sex. All participants had preserved left ventricular systolic function (mean EF 59.7% ± 6.5%). Preoperative operative risk was low to intermediate, with a mean Society of Thoracic Surgeons Predicted Risk of Mortality^18^ of 1.21% ± 1.61%.

**Table 1:** Baseline Characteristics of Participants Randomized to Routine Versus Selective Intraoperative Transesophageal Echocardiography

	TEE-on-Demand (n = 20)	TEE-by-Default (n = 20)
Age (years)	63.45 (±9.34)	67.40 (±9.07)
Sex		
Male	17 (85.0%)	18 (90.0%)
Female	3 (15.0%)	2 (10.0%)
Race		
White	15 (75.0%)	18 (90.0%)
Black/African American	2 (10.0%)	1 (5.0%)
Hawaiian/Pacific Islander	1 (5.0%)	0 (0%)
Other/Mixed	2 (10.0%)	1 (5.0%)
Ethnicity		
Hispanic/Latino	2 (10.0%)	2 (10.0%)
Not Hispanic/Latino	18 (90.0%)	18 (90.0%)
BMI (kg/m^2^)	29.44 (±5.28)	33.38 (±6.53)
ASA physical status		
3	10 (50.0%)	4 (20.0%)
4	10 (50.0%)	16 (80.0%)
EF^a^	60.15 (±6.51)	59.16 (±6.40)
Preoperative serum creatinine (mg/dL)	0.98 (±0.23)	1.04 (±0.19)
Hypertension	16 (80.0%)	18 (90.0%)
COPD	1 (5.0%)	2 (10.0%)
Left main coronary disease	9 (45.0%)	9 (45.0%)
NSTEMI	6 (30.0%)	4 (20.0%)
Number of coronaries bypassed		
1–2 vessels	2 (10.0%)	3 (15.0%)
3 vessels	12 (60.0%)	10 (50.0%)
≥4 vessels	6 (30.0%)	7 (35.0%)
STS preoperative predicted mortality risk	0.95 (±1.06)	1.46 (±2.02)
STS preoperative predicted morbidity or mortality risk	5.04 (±2.84)	7.23 (±8.69)

Values are mean (±SD) or n (%).

ASA = American Society of Anesthesiologists; BMI = body mass index; COPD = chronic obstructive pulmonary disease; EF = ejection fraction; NSTEMI = non–ST-segment elevation myocardial infarction; STS = Society of Thoracic Surgeons.

a Preoperative transthoracic echocardiography–derived ejection fraction was available for 39 of 40 patients. In one patient, normal left ventricular systolic function was confirmed by the operating surgeon based on an outside echocardiography report not available in the electronic medical record.

### Recruitment and patient equipoise

**Figure 2:**
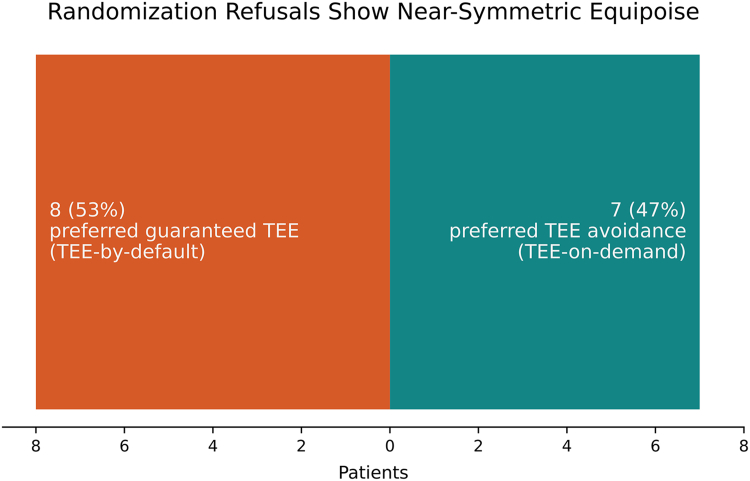
**Randomization Refusals Illustrate Patient-Level Equipoise** Distribution of stated preferences among patients who declined participation because of unwillingness to accept randomization (n = 15). Preferences were nearly evenly divided between those favoring guaranteed intraoperative transesophageal echocardiography (TEE-by-default) (8/15; 53%) and those preferring to avoid TEE unless clinically indicated (TEE-on-demand) (7/15; 47%), supporting the presence of patient-level equipoise. Abbreviations as in Figure 1.

Among 72 patients approached, 26 (36%) declined participation. Among those who declined and provided a reason, reluctance to accept randomization was the most common (n = 15). Of the fifteen patients unwilling to accept randomization, preferences were nearly evenly distributed between those favoring guaranteed TEE (8/15; 53%) and those preferring to avoid TEE unless clinically indicated (7/15; 47%) (Figure 2). This near-symmetric distribution supports the presence of patient-level equipoise within contemporary CABG practice.

### Protocol adherence and triggered TEE rate

Overall protocol adherence was high (39/40; 98%). One participant randomized to TEE-on-demand received TEE due to an allocation error, which was classified as a protocol deviation.

In the TEE-by-default arm, all 20 participants underwent successful probe placement with completion of prespecified intraoperative TEE examinations. In the TEE-on-demand arm, 16 of 20 participants (16/20; 80%) were managed without planned probe placement. Intraoperative TEE was performed in 4 participants (4/20; 20%), including 3 examinations triggered by intraoperative clinical judgment (3/20; 15%) and 1 due to the allocation error described previously. The three triggered TEEs were protocol-consistent and did not constitute crossovers or protocol deviations, as selective intraoperative TEE use was permitted by design in the TEE-on-demand arm.

### Exploratory clinical outcomes

Prespecified exploratory clinical outcomes, including postoperative creatinine, lactate, duration of mechanical ventilation, and days alive and out of hospital at 90 days, are summarized descriptively in Supplemental Table 2. No deaths or aborted procedures occurred within the 90-day follow-up time period.

Exploratory patient-reported gastrointestinal symptoms assessed within 24 and 48 hours of surgery are summarized in Supplemental Table 3. Consistent with the feasibility design and limited sample size, these analyses were descriptive and not intended to detect meaningful differences or support definitive between-group inference.

### Adverse events and safety monitoring

An independent Data and Safety Monitoring Board provided oversight throughout the trial and reviewed enrollment, feasibility metrics, and all reported adverse events. Three serious adverse events were adjudicated by the DSMB as related to intraoperative TEE exposure. These included 1 intraoperative conversion from isolated CABG to combined CABG with mitral valve replacement after TEE identified previously unrecognized severe mitral regurgitation; 1 case of esophageal bleeding requiring therapeutic endoscopic cauterization; and 1 case of severe postoperative swallowing dysfunction requiring percutaneous endoscopic gastrostomy placement. The patient who needed a percutaneous endoscopic gastrostomy subsequently died from aspiration pneumonia more than 6 months (185 days) after the index CABG procedure.

No other adverse events were adjudicated as related to study participation or the randomized intervention. Common postoperative complications, including atrial fibrillation and transient renal dysfunction, occurred at expected frequencies for isolated CABG surgery. The DSMB identified no safety concerns and recommended no protocol modifications.

### Post hoc observations to inform multicenter trial design

Several operational observations emerged during trial conduct that may inform planning for a future multicenter study. Recruitment during preoperative clinic visits was associated with higher participation than inpatient recruitment. In addition, randomization after induction of anesthesia and confirmation of hemodynamic stability was feasible within routine clinical workflow and was associated with high protocol adherence. Together, these observations may assist in refining trial procedures during multicenter implementation.

## Discussion

This randomized feasibility trial demonstrated that enrollment and randomization to alternative intraoperative TEE strategies during isolated CABG are feasible within routine clinical practice. Among 72 eligible patients approached, 46 (64%) consented to participate and 40 (56%) were successfully randomized, with high protocol adherence (39/40; 98%) and limited triggered TEE use (3/20; 15%) in the TEE-on-demand arm. The 64% consent rate was considered acceptable for a peri-operative randomized trial conducted shortly before major cardiac surgery and supports the feasibility of enrollment in a definitive trial. Importantly, among patients who declined participation because of treatment preference, preferences were nearly evenly divided between guaranteed TEE and avoidance of TEE, suggesting that nonparticipation did not reflect a uniform preference against either strategy. Implementation across multiple surgeons and anesthesiologists within a single health system indicates that clinician and patient equipoise can be operationalized, supporting the scalability of this design to a multicenter trial.

Although intraoperative TEE is associated with improved outcomes in valve and proximal aortic surgery,^4^^,^^5^ its role in isolated CABG remains uncertain.^6^, ^7^, ^8^, ^9^, ^10^ Observational studies have reported inconsistent associations with clinical outcomes,^6^, ^7^, ^8^, ^9^, ^10^ and substantial variations in practice persist across institutions and clinicians.^15^^,^^16^ This uncertainty provides the clinical foundation for evaluating whether routine or selective TEE use is the optimal strategy during isolated CABG.

Within this context of persistent clinical uncertainty,^6^, ^7^, ^8^, ^9^, ^10^ protocol adherence was high, and TEE use in the TEE-on-demand arm was limited to cases in which the treating clinicians judged it clinically necessary. The low frequency of triggered TEE and absence of discretionary crossover beyond clinician-judged clinical necessity indicate that treating surgeons and anesthesiologists were willing to adhere to assigned strategies and provide evidence of clinician-level equipoise. Participation spanned multiple surgeons and the majority of anesthesiology faculty within the health system, indicating broad clinician engagement in trial conduct. Together, these findings demonstrate that a selective TEE strategy can preserve access to TEE when clinically indicated without mandating routine use in all isolated CABG surgeries. Notably, successful enrollment and protocol adherence in 2 academic centers with high baseline TEE use suggest that equipoise-based randomization is feasible even in settings with entrenched imaging practices, supporting multicenter scalability.

Although the central question in this trial was whether TEE should be used routinely or selectively, the broader risk-benefit framework also includes recognition that TEE has anatomic visualization limitations in specific diagnostic contexts. These include incomplete visualization of the left pulmonary artery in the evaluation of pulmonary embolism^19^^,^^20^ and acoustic shadowing from the trachea and left mainstem bronchus, which creates a blind spot in the distal ascending aorta and proximal aortic arch.^21^^,^^22^ In selected settings, alternative intraoperative imaging approaches may provide complementary diagnostic information. Comprehensive epicardial echocardiography may provide diagnostic information when TEE is contraindicated or not feasible, and professional society recommendations describe standardized approaches for performing these examinations.^1^^,^^14^^,^^23^ Intracardiac echocardiography is also evolving in selected cardiovascular procedures,^24^^,^^25^ although its role in isolated CABG remains undefined.^13^^,^^14^^,^^26^ Accordingly, these modalities may be relevant in selected clinical scenarios, whereas the present feasibility trial focused specifically on randomization between TEE-by-default and TEE-on-demand strategies in isolated CABG. A future multicenter trial can further characterize the circumstances in which TEE-on-demand is triggered and how intraoperative imaging decisions are made across diverse practice settings.

Clinically meaningful safety events were observed during the trial, underscoring the importance of evaluating both potential benefit and harm in a definitive trial. The safety profile of intraoperative TEE in cardiac surgery remains incompletely characterized. Prior reviews have described a spectrum of oropharyngeal, esophageal, and gastric injuries related to TEE probe placement and manipulation, but reported complication rates have largely been derived from retrospective series relying on clinically apparent, symptom-triggered ascertainment.^27^, ^28^, ^29^, ^30^ Prospective endoscopic studies in other cardiovascular settings suggest that subclinical mucosal injury may be substantially more common than clinically recognized complications, particularly with prolonged probe manipulation and systemic anticoagulation.^11^^,^^12^ These data are relevant to isolated CABG, where the incremental benefit of TEE as an invasive hemodynamic monitor remains uncertain for many patients.^6^, ^7^, ^8^, ^9^^,^^31^

In the present trial, 2 serious esophageal complications occurred among patients exposed to TEE, including 1 case of severe postoperative swallowing dysfunction requiring gastrostomy placement and subsequent death from aspiration pneumonia approximately 6 months after surgery. Conversely, intraoperative TEE identified previously unrecognized severe mitral regurgitation in 1 patient, prompting conversion to combined CABG and mitral valve replacement. This case illustrates the potential diagnostic value of intraoperative TEE when imaging reveals clinically actionable pathology not fully appreciated before surgery. In isolated CABG, such findings may influence surgical planning, hemodynamic management, assessment of ventricular function, or evaluation of new wall-motion abnormalities after revascularization.^13^^,^^14^^,^^30^ However, the frequency with which routine TEE identifies findings that meaningfully change management, and whether these changes improve patient outcomes, remains uncertain.^9^^,^^30^ Together, these events illustrate the central clinical tension in isolated CABG: potential diagnostic benefit must be balanced against procedure-related harm. Because this feasibility trial was not powered to estimate clinical event rates, a larger multicenter randomized trial with prospective, standardized ascertainment of TEE-related adverse events will be required to quantify the comparative benefits and harms of TEE-by-default vs TEE-on-demand strategies, while also identifying patient-, procedural-, and imaging-related factors associated with TEE-related complications.

### Study limitations

This study has limitations inherent to its design as a randomized feasibility trial with a small sample size conducted at 2 hospitals within a single health system. First, the trial was not designed or powered to detect differences in clinical outcomes, and any observed differences in exploratory endpoints should be interpreted only as hypothesis-generating. The limited sample size also restricts the precision of effect estimates and precludes meaningful subgroup analyses. Second, the postoperative gastrointestinal symptom survey was not validated but was intentionally included to inform the selection and refinement of patient-centered endpoints for a future multicenter trial, including the use of a validated swallowing dysfunction instrument. Third, because blinding of the intraoperative care team was not feasible, knowledge of treatment assignment could have influenced intraoperative decision-making. Finally, conduct within a single health system may limit generalizability, although clinician participation was broad across surgeons and anesthesiology faculty.

## Conclusions

Randomization to alternative intraoperative TEE strategies during isolated CABG is feasible within contemporary clinical practice, with high protocol adherence and preserved clinician and patient equipoise. These findings support the design and conduct of a larger multicenter randomized trial to evaluate the comparative effectiveness and safety of TEE-by-default and TEE-on-demand strategies.

Perspectives **COMPETENCY IN MEDICAL KNOWLEDGE:** Intraoperative transesophageal echocardiography (TEE) is commonly used during isolated coronary artery bypass grafting despite marked practice variation, uncertain incremental benefit, and procedure-related risks borne only by patients who undergo probe placement. This pilot demonstrated that randomization to TEE-by-default versus TEE-on-demand is feasible and can achieve high protocol adherence. Participation by cardiac anesthesiologists and surgeons, together with patients' willingness to accept randomization despite differing individual preferences provides empirical evidence of clinician- and patient-level equipoise and establishes that this longstanding clinical question can be resolved in a definitive randomized trial.

**TRANSLATIONAL OUTLOOK:** An adequately powered, multicenter randomized trial is needed to determine whether TEE-by-default improves serious outcomes sufficiently to justify universal exposure or whether a TEE-on-demand strategy can preserve clinical benefit while reducing avoidable harm. The results of a multicenter trial could directly inform guidelines, reduce unwarranted practice variation, and define when intraoperative TEE adds meaningful value to patients undergoing isolated coronary artery bypass grafting surgery.

## Funding support and author disclosures

This study was funded, in part, by the 10.13039/100000002National Institutes of Health, 10.13039/100000050National Heart, Lung, and Blood Institute (K23HL166964 to Dr MacKay). Dr Szeto is a speaker for Abbott as a speaker; is a speaker, advisory board member, and investigator for Artivion, Edwards Lifesciences, Medtronic, and Terumo Aortic. Dr Giri has served as an advisor to Boston Scientific, Stryker, Medtronic, and Endovascular Engineering, with research funds paid to his institution from these companies. All other authors have reported that they have no relationships relevant to the contents of this paper to disclose.
