# Supplementary material for: Randomized Feasibility Trial of Routine Versus Selective Transesophageal Echocardiography During Isolated Coronary Artery Bypass Grafting
Source: JACC Adv. 2026 Jul 20;5(8):103030. doi: 10.1016/j.jacadv.2026.103030 (PMC13393670; doi:10.1016/j.jacadv.2026.103030)
Supplement: Supplemental data [file mmc1.pdf]

eSupplement

Randomized Feasibility Trial of Routine Versus Selective Transesophageal Echocardiography During Isolated Coronary Artery Bypass Grafting

MacKay EJ, et al

Contents

- **eTable1**. Eligibility Criteria
- **eTable 2**. Exploratory Clinical Outcomes
- **eTable 3**. Postoperative Gastrointestinal Symptoms
- **eTable 4**. Trial Investigators: Participating Surgeons and Anesthesiologists
- **eTable 5**. Trial Operational Support Personnel

eTable 1: Protocol Inclusion and Exclusion Criteria

Category	Criterion
**Inclusion Criteria**	Scheduled to undergo isolated coronary artery bypass graft (CABG) surgery at a hospital within the University of Pennsylvania Health System
	Age ≥18 years
	Left ventricular ejection fraction of 50% or greater
	Transthoracic echocardiography performed within 1 year prior to CABG surgery
	Left heart catheterization performed within 1 year prior to CABG surgery
	English language fluency or communication facilitated by a certified language interpreter
	Ability to provide informed consent in English or via a language interpreter
	Willingness to comply with all study procedures
**Exclusion Criteria**	Documented moderate or greater valvular heart disease (aortic, mitral, tricuspid, or pulmonic stenosis or regurgitation)
	Planned CABG surgery with "possible" or "definite" aortic intervention listed
	Planned CABG surgery with "possible" or "definite" valve repair or replacement listed
	Prior cardiac surgery (ie, scheduled with a "redo" modifier)
	Proximal or critical left main coronary artery disease (eg, ≥90% stenosis)
	Preexisting anomalous coronary arteries
	End-stage renal disease requiring hemodialysis
	Chronic kidney disease stage 3, 4, or 5
	Stroke with residual focal neurologic deficits within 90 days prior to surgery
	Presence of any presurgical mechanical circulatory support device, including intra-aortic balloon pump, percutaneous right ventricular assist device, Impella, or extracorporeal membrane oxygenation
	Absolute contraindication to transesophageal echocardiography, including prior esophagectomy, esophagogastrectomy, or documented esophageal trauma
	Relative contraindication to transesophageal echocardiography, including esophageal varices, prior gastric bypass surgery, descending thoracic aortic aneurysm, or oropharyngeal anatomic abnormalities
	Severe pulmonary hypertension, defined as pulmonary arterial pressure ≥60 mm Hg or pulmonary vascular resistance ≥3 Wood units
	Hemodynamic instability after induction of anesthesia and endotracheal intubation, defined by any of the following: placement of an intra-aortic balloon pump; initiation of venoarterial extracorporeal membrane oxygenation; placement of right- or left-sided percutaneous mechanical circulatory support; initiation of epinephrine infusion ≥0.08 μg/kg/min for ≥5 minutes; initiation of norepinephrine infusion ≥0.10 μg/kg/min for ≥5 minutes; initiation of phenylephrine infusion ≥1.5 μg/kg/min for ≥5 minutes; or initiation of vasopressin infusion ≥0.04 units/min for ≥5 minutes

eTable 2. Exploratory Clinical Outcomes by Randomized Arm

Outcome[a]	Total (n=40)	TEE-on-demand (n=20)	TEE-by-default (n=20)
Postoperative creatinine at 24 h (mg/dL), mean ± SD [95% CI]	0.88 ± 0.23 [0.81–0.95]	0.85 ± 0.21 [0.75–0.94]	0.92 ± 0.25 [0.81–1.03]
Postoperative lactate (mmol/L), mean ± SD [95% CI]	1.87 ± 1.98 [1.25–2.48]	1.45 ± 0.77 [1.11–1.78]	2.29 ± 2.67 [1.12–3.46]
Postoperative ventilator duration during index hospitalization (hours), mean ± SD [95% CI]	5.62 ± 3.97 [4.39–6.85]	5.16 ± 4.26 [3.29–7.03]	6.08 ± 3.70 [4.46–7.70]
Days alive and out of hospital at 90 days, mean ± SD [95% CI]	81.97 ± 8.34 [79.39–84.56]	83.25 ± 7.73 [79.86–86.64]	80.70 ± 8.92 [76.79–84.61]

[a] Continuous variables are presented as mean ± standard deviation with 95% confidence intervals. Categorical variables are presented as counts and percentages. Analyses are descriptive and not powered for definitive between-group comparisons.
Abbreviations: DAOH = days alive and out of hospital.

eTable 3. Exploratory Postoperative Gastrointestinal Symptoms at 24 and 48 Hours by Randomized Group

Symptom[a]	TEE-on-demand, 24 h (n=20)	TEE-by-default, 24 h (n=20)	TEE-on-demand, 48 h (n=20)	TEE-by-default, 48 h (n=20)
Sore throat	4 (20.0%)	5 (25.0%)	6 (30.0%)	6 (30.0%)
Dry throat	8 (40.0%)	10 (50.0%)	10 (50.0%)	6 (30.0%)
Swallowing difficulty	2 (10.0%)	3 (15.0%)	2 (10.0%)	3 (15.0%)
Painful swallowing	3 (15.0%)	4 (20.0%)	2 (10.0%)	3 (15.0%)
Nausea or vomiting	4 (20.0%)	7 (35.0%)	4 (20.0%)	4 (20.0%)
Gastroesophageal reflux symptoms	1 (5.0%)	4 (20.0%)	3 (15.0%)	3 (15.0%)
Taste of blood[b]	1 (5.0%)	0 (<1%)	—	—

[a] Symptoms were assessed using nonvalidated binary patient-reported items and are presented descriptively. Values are No. (%).
[b] No participants reported taste of blood at 48 hours after surgery.
[c] Denominators reflect the number of participants who completed the GI–1 and GI–2 surveys (GI–1: 38/40; GI–2: 40/40). Missing data were not imputed.

eTable 4. ECHO Pilot Trial Investigators and Roles

Investigator Name	Degree	Role
Lourdes F. Al Ghofaily	MD	Anesthesiologist
Yianni Augoustides	MD	Anesthesiologist
Maurer Biscotti	MD	Surgeon
Chase Brown	MD, MS	Surgeon
Alessandra I. Cardi	MD	Anesthesiologist
Marisa Cevasco	MD, MPH	Surgeon
Navdeep K. Chahal	MD	Anesthesiologist
Joseph A. Colao	MD	Anesthesiologist
Holly E. Corkill	MD	Anesthesiologist
Jared Feinman	MD	Anesthesiologist
Emily Gordon	MD	Anesthesiologist
Jacob Gutsche	MD	Anesthesiologist
Michael Ibrahim	MD, PhD	Surgeon
Jiri Horak	MD	Anesthesiologist
Zev Noah Kornfield	MD	Anesthesiologist
Jeremy Kukafka	MD	Anesthesiologist
Kendall Lawrence	MD	Surgeon
Regina E. Linganna	MD	Anesthesiologist
Emily J. MacKay	DO, MS	Anesthesiologist
Tal Mandelbaum	MD	Anesthesiologist
Bonnie Milas	MD	Anesthesiologist
Saumil Patel	MD	Anesthesiologist
Stuart M. Sacks	MD	Anesthesiologist
Joseph Savino	MD	Anesthesiologist
Ronak Shah	MD	Anesthesiologist
Audrey Spelde	MD	Anesthesiologist
Wilson Y. Szeto	MD	Surgeon
Nabil Thalji	MD, PhD	Anesthesiologist
Asad A. Usman	MD	Anesthesiologist
William Vernick	MD	Anesthesiologist
Kelvin Wang	MD	Anesthesiologist
Stuart J. Weiss	MD, PhD	Anesthesiologist
Elizabeth Zhou	MD	Anesthesiologist

eTable 5. ECHO Pilot Trial Study Team and Operational Support

Team Member Name	Role
Kelli Cook	Research Coordinator
Destiny T. Garcia	Research Coordinator
Marisa Konig	Lead Research Coordinator
Zane Mazur	Research Coordinator
Waleed Mujib	Medical Student (Perelman SOM)
Amber M. Stefanowicz	Research Coordinator
Karah Whatley	Project Manager
